# *N*′-[(*E*)-(1-Methyl-1*H*-pyrrol-2-yl)methyl­idene]benzohydrazide

**DOI:** 10.1107/S1600536810025390

**Published:** 2010-07-03

**Authors:** Abid Hussain, Zahid Shafiq, M. Nawaz Tahir, Muhammad Yaqub

**Affiliations:** a Department of Chemistry, Bahauddin Zakariya University, Multan 60800, Pakistan; b Department of Physics, University of Sargodha, Sargodha, Pakistan

## Abstract

In the title compound, C_13_H_13_N_3_O, the phenyl and pyrrole rings are inclined at 47.45 (8)°. In the crystal, inter­molecular N—H⋯O and C—H⋯O hydrogen bonds form *R*_2_^1^(6) ring motifs. Mol­ecules connected through these hydrogen bonds are arranged into polymeric chains extending along the *c* axis.

## Related literature

For related structures, see: Shafiq *et al.* (2009*a* ▶,*b* ▶). For graph-set notation, see: Bernstein *et al.* (1995 ▶).
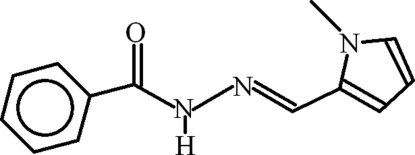


## Experimental

### 

#### Crystal data

C_13_H_13_N_3_O
*M* *_r_* = 227.26
Monoclinic, 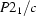

*a* = 11.1170 (8) Å
*b* = 11.6329 (9) Å
*c* = 9.6735 (6) Å
β = 110.241 (3)°
*V* = 1173.75 (15) Å^3^
*Z* = 4
Mo *K*α radiation
μ = 0.09 mm^−1^
*T* = 296 K
0.28 × 0.12 × 0.10 mm

#### Data collection

Bruker Kappa APEXII CCD diffractometer
Absorption correction: multi-scan (*SADABS*; Bruker, 2005 ▶) *T*_min_ = 0.942, *T*_max_ = 0.952
11164 measured reflections
2930 independent reflections
1770 reflections with *I* > 2σ(*I*)
*R*_int_ = 0.047

#### Refinement

*R*[*F* ^2^ > 2σ(*F* ^2^)] = 0.052
*wR*(*F* ^2^) = 0.139
*S* = 1.02
2930 reflections
155 parameters
H-atom parameters constrained
Δρ_max_ = 0.19 e Å^−3^
Δρ_min_ = −0.17 e Å^−3^

### 

Data collection: *APEX2* (Bruker, 2009 ▶); cell refinement: *SAINT* (Bruker, 2009 ▶); data reduction: *SAINT*; program(s) used to solve structure: *SHELXS97* (Sheldrick, 2008 ▶); program(s) used to refine structure: *SHELXL97* (Sheldrick, 2008 ▶); molecular graphics: *ORTEP-3 for Windows* (Farrugia, 1997 ▶) and *PLATON* (Spek, 2009 ▶); software used to prepare material for publication: *WinGX* (Farrugia, 1999 ▶) and *PLATON*.

## Supplementary Material

Crystal structure: contains datablocks global, I. DOI: 10.1107/S1600536810025390/gk2289sup1.cif

Structure factors: contains datablocks I. DOI: 10.1107/S1600536810025390/gk2289Isup2.hkl

Additional supplementary materials:  crystallographic information; 3D view; checkCIF report

## Figures and Tables

**Table 1:** Hydrogen-bond geometry (Å, °)

*D*—H⋯*A*	*D*—H	H⋯*A*	*D*⋯*A*	*D*—H⋯*A*
N1—H1⋯O1^i^	0.86	2.11	2.910 (2)	155
C8—H8⋯O1^i^	0.93	2.38	3.209 (2)	148

Symmetry code: (i) 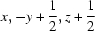.

**Fig. 1.**
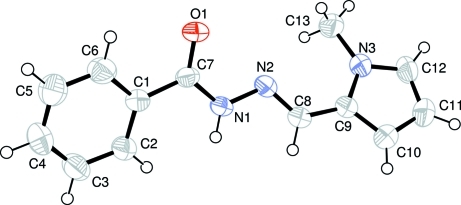
View of the title compound with the atom numbering scheme. The displacement ellipsoids are drawn at the 50% probability level. H atoms are shown by small circles of arbitrary radii.

**Fig. 2.**
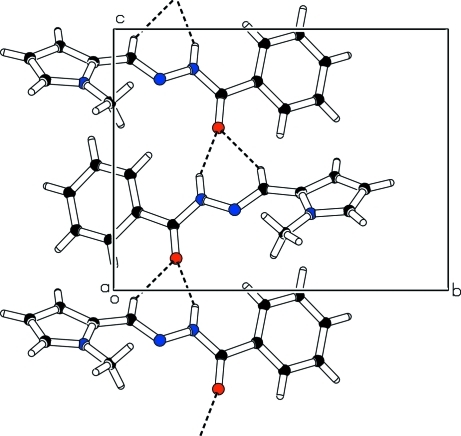
The partial packing (PLATON; Spek, 2009) showing one dimensional polymeric chain via hydrogen bonds. Hydrogen bonds are shown as dashed lines.

## supplementary crystallographic information

### Comment

We have reported crystal structures of Schiff bases containing benzohydrazide (Shafiq *et al.*, 2009*a*, 2009*b*) and as a part of this project, we report herein the structure and synthesis of the title compound (I, Fig. 1).

In (I) the group A (C1–C7/N1/N2) of benzohydrazide and group B (C8—C12/N3/C13) of 1-methyl-1*H*-pyrrole are planar with r. m. s. deviation of 0.0345 and 0.0249 Å, respectively. The O atom of the carbonyl group is at a distance of 0.1993 (22) Å from the mean square plane of A. The dihedral angle between A/B is 46.78 (7)°. There exist intermolecular hydrgen bonds of N—H···O and C—H···O types (Table 1) that complete *R*_2_^1^(6) ring motif (Bernstein *et al.*, 1995). The molecules are stabilized in the form of one dimensional polymeric chains (Fig. 2) extending along the crystallographic *c* axis.

### Experimental

To a hot stirred solution of benzohydrazide (1.36 g, 0.01 mole) in ethanol 15 ml was added *N*-methylpyrrol-2-carboxaldehyde (1.1 ml, 0.01 mol). The resultant mixture was then heated under reflux. The reaction was monitored through TLC. After an hour, the precipitate was formed. The reaction mixture was further heated for 30 min. The resultant crude material was recrystallized from methanol to afford colourless needles of the titke compound.

### Refinement

The H atoms were positioned geometrically (N–H = 0.86 Å, C–H = 0.93–0.96 Å) and refined as riding with *U*_iso_(H) = *xU*_eq_(C, N), where *x* = 1.5 for methyl group and *x* = 1.2 for all other H atoms.

### Crystal data

**Table:**

C_13_H_13_N_3_O	*F*(000) = 480
*M**_r_* = 227.26	*D*_x_ = 1.286 Mg m^−^^3^
Monoclinic, *P*2_1_/*c*	Mo *K*α radiation, λ = 0.71073 Å
Hall symbol: -P 2ybc	Cell parameters from 1770 reflections
*a* = 11.1170 (8) Å	θ = 2.6–28.4°
*b* = 11.6329 (9) Å	µ = 0.09 mm^−^^1^
*c* = 9.6735 (6) Å	*T* = 296 K
β = 110.241 (3)°	Needle, colorless
*V* = 1173.75 (15) Å^3^	0.28 × 0.12 × 0.10 mm
*Z* = 4	

### Data collection

**Table:**

Bruker Kappa APEXII CCD diffractometer	2930 independent reflections
Radiation source: fine-focus sealed tube	1770 reflections with *I* > 2σ(*I*)
graphite	*R*_int_ = 0.047
Detector resolution: 7.50 pixels mm^-1^	θ_max_ = 28.4°, θ_min_ = 2.6°
ω scans	*h* = −14→14
Absorption correction: multi-scan (*SADABS*; Bruker, 2005)	*k* = −15→15
*T*_min_ = 0.942, *T*_max_ = 0.952	*l* = −12→9
11164 measured reflections	

### Refinement

**Table:**

Refinement on *F*^2^	Primary atom site location: structure-invariant direct methods
Least-squares matrix: full	Secondary atom site location: difference Fourier map
*R*[*F*^2^ > 2σ(*F*^2^)] = 0.052	Hydrogen site location: inferred from neighbouring sites
*wR*(*F*^2^) = 0.139	H-atom parameters constrained
*S* = 1.02	*w* = 1/[σ^2^(*F*_o_^2^) + (0.0555*P*)^2^ + 0.160*P*] where *P* = (*F*_o_^2^ + 2*F*_c_^2^)/3
2930 reflections	(Δ/σ)_max_ < 0.001
155 parameters	Δρ_max_ = 0.19 e Å^−^^3^
0 restraints	Δρ_min_ = −0.16 e Å^−^^3^

### Special details

**Table:**

Geometry. Bond distances, angles *etc*. have been calculated using the rounded fractional coordinates. All su's are estimated from the variances of the (full) variance-covariance matrix. The cell e.s.d.'s are taken into account in the estimation of distances, angles and torsion angles
Refinement. Refinement of *F*^2^ against ALL reflections. The weighted *R*-factor *wR* and goodness of fit *S* are based on *F*^2^, conventional *R*-factors *R* are based on *F*, with *F* set to zero for negative *F*^2^. The threshold expression of *F*^2^ > σ(*F*^2^) is used only for calculating *R*-factors(gt) *etc*. and is not relevant to the choice of reflections for refinement. *R*-factors based on *F*^2^ are statistically about twice as large as those based on *F*, and *R*- factors based on ALL data will be even larger.

### Fractional atomic coordinates and isotropic or equivalent isotropic displacement parameters (Å^2^)

**Table:**

	*x*	*y*	*z*	*U*_iso_*/*U*_eq_	
O1	0.71496 (14)	0.18682 (11)	0.12223 (13)	0.0554 (5)	
N1	0.75685 (15)	0.26311 (12)	0.34764 (15)	0.0431 (5)	
N2	0.80816 (15)	0.36186 (12)	0.30879 (16)	0.0442 (5)	
N3	0.93230 (15)	0.58927 (13)	0.29335 (16)	0.0457 (5)	
C1	0.66632 (16)	0.07206 (14)	0.29906 (18)	0.0394 (5)	
C2	0.67749 (19)	0.05014 (16)	0.4433 (2)	0.0492 (7)	
C3	0.6323 (2)	−0.05186 (18)	0.4795 (2)	0.0602 (8)	
C4	0.5752 (2)	−0.13174 (18)	0.3738 (3)	0.0639 (8)	
C5	0.5635 (2)	−0.11093 (19)	0.2302 (3)	0.0695 (9)	
C6	0.6095 (2)	−0.01047 (18)	0.1927 (2)	0.0565 (7)	
C7	0.71456 (16)	0.17851 (14)	0.24946 (19)	0.0389 (6)	
C8	0.80671 (18)	0.44929 (15)	0.38773 (19)	0.0454 (6)	
C9	0.85533 (18)	0.56059 (15)	0.37314 (19)	0.0445 (6)	
C10	0.8295 (2)	0.66046 (17)	0.4330 (2)	0.0592 (8)	
C11	0.8898 (2)	0.75049 (18)	0.3880 (3)	0.0657 (9)	
C12	0.9521 (2)	0.70430 (16)	0.3038 (2)	0.0563 (7)	
C13	0.9916 (2)	0.51144 (17)	0.2185 (2)	0.0562 (8)	
H1	0.75240	0.25659	0.43429	0.0517*	
H2	0.71552	0.10422	0.51611	0.0591*	
H3	0.64097	−0.06633	0.57704	0.0723*	
H4	0.54425	−0.19993	0.39907	0.0767*	
H5	0.52429	−0.16507	0.15793	0.0833*	
H6	0.60254	0.00238	0.09532	0.0679*	
H8	0.77103	0.43980	0.46091	0.0545*	
H10	0.78019	0.66688	0.49312	0.0711*	
H11	0.88753	0.82779	0.41155	0.0788*	
H12	1.00108	0.74519	0.25975	0.0676*	
H13A	1.05186	0.55310	0.18654	0.0843*	
H13B	0.92678	0.47841	0.13455	0.0843*	
H13C	1.03550	0.45137	0.28485	0.0843*	

### Atomic displacement parameters (Å^2^)

**Table:**

	*U*^11^	*U*^22^	*U*^33^	*U*^12^	*U*^13^	*U*^23^
O1	0.0848 (10)	0.0528 (8)	0.0376 (7)	−0.0061 (7)	0.0325 (7)	−0.0043 (6)
N1	0.0592 (10)	0.0417 (8)	0.0346 (8)	−0.0077 (7)	0.0241 (7)	−0.0013 (6)
N2	0.0558 (10)	0.0409 (8)	0.0399 (8)	−0.0051 (7)	0.0216 (7)	0.0028 (7)
N3	0.0516 (9)	0.0408 (8)	0.0479 (9)	−0.0001 (7)	0.0214 (8)	0.0031 (7)
C1	0.0378 (9)	0.0440 (10)	0.0371 (9)	0.0005 (8)	0.0138 (8)	−0.0010 (8)
C2	0.0590 (12)	0.0476 (11)	0.0426 (11)	−0.0056 (9)	0.0195 (9)	−0.0009 (8)
C3	0.0711 (14)	0.0606 (13)	0.0520 (12)	−0.0075 (11)	0.0251 (11)	0.0097 (10)
C4	0.0647 (14)	0.0540 (13)	0.0751 (16)	−0.0136 (10)	0.0269 (12)	0.0061 (11)
C5	0.0750 (16)	0.0641 (14)	0.0675 (15)	−0.0270 (12)	0.0224 (12)	−0.0157 (12)
C6	0.0604 (13)	0.0633 (13)	0.0446 (11)	−0.0146 (10)	0.0165 (10)	−0.0064 (10)
C7	0.0418 (10)	0.0430 (10)	0.0351 (9)	0.0039 (8)	0.0175 (8)	−0.0013 (8)
C8	0.0568 (12)	0.0453 (11)	0.0388 (10)	−0.0008 (8)	0.0224 (9)	0.0022 (8)
C9	0.0548 (11)	0.0421 (10)	0.0384 (10)	0.0003 (8)	0.0185 (9)	0.0027 (8)
C10	0.0787 (15)	0.0468 (11)	0.0624 (13)	0.0016 (10)	0.0375 (12)	−0.0031 (10)
C11	0.0869 (17)	0.0394 (11)	0.0789 (16)	−0.0007 (10)	0.0391 (14)	−0.0016 (10)
C12	0.0662 (13)	0.0407 (11)	0.0668 (14)	−0.0041 (9)	0.0291 (11)	0.0076 (9)
C13	0.0645 (14)	0.0516 (12)	0.0625 (13)	−0.0037 (10)	0.0346 (11)	−0.0038 (10)

### Geometric parameters (Å, °)

**Table:**

O1—C7	1.236 (2)	C9—C10	1.372 (3)
N1—N2	1.391 (2)	C10—C11	1.392 (3)
N1—C7	1.335 (2)	C11—C12	1.350 (3)
N2—C8	1.275 (2)	C2—H2	0.9300
N3—C9	1.377 (3)	C3—H3	0.9300
N3—C12	1.354 (2)	C4—H4	0.9300
N3—C13	1.452 (3)	C5—H5	0.9300
N1—H1	0.8600	C6—H6	0.9300
C1—C2	1.381 (2)	C8—H8	0.9300
C1—C7	1.493 (2)	C10—H10	0.9300
C1—C6	1.389 (3)	C11—H11	0.9300
C2—C3	1.380 (3)	C12—H12	0.9300
C3—C4	1.365 (3)	C13—H13A	0.9600
C4—C5	1.371 (4)	C13—H13B	0.9600
C5—C6	1.374 (3)	C13—H13C	0.9600
C8—C9	1.429 (3)		
			
O1···N2	2.6801 (19)	C13···H11^iii^	3.0200
O1···N1^i^	2.910 (2)	H1···C2	2.5500
O1···C8^i^	3.209 (2)	H1···H2	2.0400
O1···H6	2.4500	H1···H8	2.1500
O1···H1^i^	2.1100	H1···O1^ii^	2.1100
O1···H2^i^	2.6400	H2···N1	2.6100
O1···H8^i^	2.3800	H2···H1	2.0400
N1···O1^ii^	2.910 (2)	H2···O1^ii^	2.6400
N2···N3	3.011 (2)	H2···N2^ii^	2.6900
N2···O1	2.6801 (19)	H2···H13B^ii^	2.4300
N2···C13	3.030 (3)	H3···C9^ii^	3.0300
N3···N2	3.011 (2)	H4···C7^ix^	3.0800
N1···H2	2.6100	H6···O1	2.4500
N2···H13B	2.8200	H6···C6^vii^	2.9600
N2···H12^iii^	2.7800	H6···H6^vii^	2.3900
N2···H2^i^	2.6900	H8···H1	2.1500
N2···H13C	2.8200	H8···O1^ii^	2.3800
C3···C3^iv^	3.328 (3)	H11···C13^vi^	3.0200
C8···O1^ii^	3.209 (2)	H12···H13A	2.4700
C9···C9^v^	3.591 (3)	H12···N2^vi^	2.7800
C11···C13^vi^	3.599 (3)	H12···H13C^vi^	2.4500
C13···C11^iii^	3.599 (3)	H13A···H12	2.4700
C13···N2	3.030 (3)	H13A···C1^vi^	3.1000
C1···H13A^iii^	3.1000	H13A···C7^vi^	2.8500
C2···H13B^ii^	2.7700	H13B···N2	2.8200
C2···H1	2.5500	H13B···C2^i^	2.7700
C6···H6^vii^	2.9600	H13B···H2^i^	2.4300
C7···H13A^iii^	2.8500	H13C···N2	2.8200
C7···H4^viii^	3.0800	H13C···C8	3.0400
C8···H13C	3.0400	H13C···C12^iii^	3.0200
C9···H3^i^	3.0300	H13C···H12^iii^	2.4500
C10···H13C^v^	2.9300	H13C···C10^v^	2.9300
C12···H13C^vi^	3.0200		
			
N2—N1—C7	119.54 (14)	C1—C2—H2	120.00
N1—N2—C8	113.93 (16)	C3—C2—H2	120.00
C9—N3—C12	108.30 (16)	C2—C3—H3	120.00
C9—N3—C13	127.28 (16)	C4—C3—H3	120.00
C12—N3—C13	124.28 (17)	C3—C4—H4	120.00
N2—N1—H1	120.00	C5—C4—H4	120.00
C7—N1—H1	120.00	C4—C5—H5	120.00
C6—C1—C7	117.20 (15)	C6—C5—H5	120.00
C2—C1—C6	118.66 (17)	C1—C6—H6	120.00
C2—C1—C7	124.13 (16)	C5—C6—H6	120.00
C1—C2—C3	120.11 (17)	N2—C8—H8	117.00
C2—C3—C4	120.73 (19)	C9—C8—H8	117.00
C3—C4—C5	119.7 (2)	C9—C10—H10	126.00
C4—C5—C6	120.3 (2)	C11—C10—H10	126.00
C1—C6—C5	120.52 (19)	C10—C11—H11	126.00
N1—C7—C1	117.39 (15)	C12—C11—H11	127.00
O1—C7—N1	121.93 (16)	N3—C12—H12	125.00
O1—C7—C1	120.68 (15)	C11—C12—H12	125.00
N2—C8—C9	125.45 (18)	N3—C13—H13A	109.00
N3—C9—C10	107.02 (16)	N3—C13—H13B	110.00
C8—C9—C10	125.74 (19)	N3—C13—H13C	110.00
N3—C9—C8	127.20 (17)	H13A—C13—H13B	109.00
C9—C10—C11	108.14 (19)	H13A—C13—H13C	109.00
C10—C11—C12	107.00 (19)	H13B—C13—H13C	109.00
N3—C12—C11	109.53 (19)		
			
C7—N1—N2—C8	158.43 (18)	C2—C1—C7—O1	171.48 (19)
N2—N1—C7—O1	−2.8 (3)	C2—C1—C7—N1	−8.4 (3)
N2—N1—C7—C1	177.08 (16)	C6—C1—C7—O1	−7.0 (3)
N1—N2—C8—C9	178.86 (18)	C6—C1—C7—N1	173.11 (18)
C12—N3—C9—C8	177.51 (19)	C1—C2—C3—C4	−0.6 (3)
C12—N3—C9—C10	−0.4 (2)	C2—C3—C4—C5	0.5 (4)
C13—N3—C9—C8	−6.7 (3)	C3—C4—C5—C6	0.3 (4)
C13—N3—C9—C10	175.43 (18)	C4—C5—C6—C1	−1.1 (4)
C9—N3—C12—C11	−0.1 (2)	N2—C8—C9—N3	−13.0 (3)
C13—N3—C12—C11	−176.05 (19)	N2—C8—C9—C10	164.5 (2)
C6—C1—C2—C3	−0.2 (3)	N3—C9—C10—C11	0.7 (2)
C7—C1—C2—C3	−178.67 (19)	C8—C9—C10—C11	−177.2 (2)
C2—C1—C6—C5	1.1 (3)	C9—C10—C11—C12	−0.7 (3)
C7—C1—C6—C5	179.6 (2)	C10—C11—C12—N3	0.5 (3)

Symmetry codes: (i) *x*, −*y*+1/2, *z*−1/2; (ii) *x*, −*y*+1/2, *z*+1/2; (iii) −*x*+2, *y*−1/2, −*z*+1/2; (iv) −*x*+1, −*y*, −*z*+1; (v) −*x*+2, −*y*+1, −*z*+1; (vi) −*x*+2, *y*+1/2, −*z*+1/2; (vii) −*x*+1, −*y*, −*z*; (viii) −*x*+1, *y*+1/2, −*z*+1/2; (ix) −*x*+1, *y*−1/2, −*z*+1/2.

### Hydrogen-bond geometry (Å, °)

**Table:**

*D*—H···*A*	*D*—H	H···*A*	*D*···*A*	*D*—H···*A*
N1—H1···O1^ii^	0.86	2.11	2.910 (2)	155
C8—H8···O1^ii^	0.93	2.38	3.209 (2)	148

Symmetry codes: (ii) *x*, −*y*+1/2, *z*+1/2.
